# Repeat Next-Generation Sequencing (15-Gene Panel) in Unifocal, Synchronous, and Metachronous Non-Small-Cell Lung Cancer—A Single-Center Experience

**DOI:** 10.3390/curroncol31080334

**Published:** 2024-08-03

**Authors:** Shelley Kuang, Kaitlin Chen, Sachin Sayal, Gajeni Prabahan, Mary R. Rabey, Lisa W. Le, Andrew Seto, Frances A. Shepherd, Geoffrey Liu, Penelope Bradbury, Adrian G. Sacher, Jennifer H. Law, Peter Sabatini, Tracy L. Stockley, Ming S. Tsao, Natasha B. Leighl

**Affiliations:** 1 Department of Medical Oncology, Princess Margaret Cancer Centre, University Health Network, Toronto, ON M5G 1M9, Canada; 2 Department of Biostatistics, University Health Network, Toronto, ON M5G 2C1, Canada; lisa.le@uhn.ca (L.W.L.);; 3 Division of Clinical Laboratory Genetics, Laboratory Medicine Program, University Health Network, Toronto, ON M5G 2C4, Canada; 4 Advanced Molecular Diagnostics Laboratory, University Health Network, Toronto, ON M5G 2M9, Canada; 5 Department of Laboratory Medicine and Pathobiology, University of Toronto, Toronto, ON M5S 1A8, Canada

**Keywords:** non-small-cell lung cancer, next generation sequencing, multifocal lung cancer, synchronous, metachronous

## Abstract

In advanced non-squamous non-small-cell lung cancer (NSCLC), routine testing with next-generation sequencing (NGS) is recommended to identify actionable genomic alterations (AGAs). The therapeutic implications of repeated NGS testing on synchronous and metachronous tumors are unclear. Between February 2017 and October 2020, NSCLC samples from a single institution were reflex-tested using a targeted 15-gene NGS panel (TruSight Tumor 15, Illumina). Thirty-eight patients were identified with multiple NGS results from 82 samples: 11% were from single unifocal, 51% were from synchronous, and 38% were from metachronous tumors. Changes in *EGFR*, *KRAS*, *PI3KCA*, and *TP53* variants were found in 22 patients’ samples (58%). No changes were seen with longitudinal testing of multiple samples from single unifocal tumors, while changes were observed in 60% of synchronous and 71% of metachronous tumors. Of these, 26% of patients had AGA differences between samples. Acknowledging the limited sample size, a significant difference in overall survival was observed between synchronous separate primaries and metastasis. Repeat NGS testing of synchronous and metachronous NSCLC tumors may identify differing variants in >50% of patients. These changes may reflect separate primary lung carcinomas, tumor heterogeneity among intrapulmonary metastases, and clonal evolution. NGS testing of multiple tumors may enhance the identification of therapeutic targets for treatment decisions.

## 1. Introduction

In advanced non-small-cell lung cancer (NSCLC), next-generation sequencing (NGS) using multigene panels is recommended by the American Society of Clinical Oncology-Ontario Health (ASCO-OH), National Comprehensive Cancer Network (NCCN), International Association for the Study of Lung Cancer/College of American Pathology (IASLC/CAP), and European Society of Medical Oncology (ESMO) guidelines for identifying actionable genomic alterations (AGAs) at the time of diagnosis [1,2,3,4]. If identified, matched targeted therapies can improve quality of life, tumor response, progression-free survival, and overall survival (OS) compared to cytotoxic chemotherapy.

With the increasing use of NGS in routine diagnostic practice, some patients may have multiple samples tested to confirm initial test results (e.g., biopsy and matched resected tumor) to distinguish between separate primary lung carcinomas and intrapulmonary metastasis, which affects staging as well as treatment decisions, and to identify potential new drivers in samples from cases of recurrence. While comprehensive morphologic analysis may identify separate primary lung carcinomas from intrapulmonary metastasis, reports suggest that genomic testing may further define these categories [5,6,7,8,9]. Multiple testing can identify differing results from synchronous or metachronous tumors as well as multiple samples from the same lesion via endobronchial ultrasound biopsies. While comprehensive NGS testing is now standard for advanced NSCLC samples, countries including Canada had delayed uptake of this technology. Prior to the funding of comprehensive NGS for lung cancer in Canada, we investigated the impact of a targeted NGS 15-gene panel (TruSight Tumor 15 [TST15], Illumina, San Diego, CA, USA) as part of the routine reflex testing for non-squamous NSCLC samples at a single institution [10]. In this report, we explore the clinical implications of NGS testing of multiple samples from patients with unifocal, synchronous, and metachronous NSCLC tumors.

## 2. Materials and Methods

The conduct of this prospective study was approved by the University Health Network (UHN) Research Ethics Board (17-5384). Between February 2017 and October 2020, the UHN Genome Diagnostics Laboratory used the TST15 gene panel to molecularly profile diagnostic samples of non-squamous NSCLC tumor tissue ordered as reflex testing by UHN pathologists or as requested by the patient’s medical oncologist. During this study period, only routine *EGFR* testing, ALK immunohistochemistry (IHC), and ROS1 IHC screening were funded standard of care in Canada. Formalin-fixed, paraffin-embedded (FFPE) tumor and cytology samples were assessed for sufficiency and tumor-rich areas identified by a board-certified pathologist, with a minimum tumor tissue surface area of 10 mm^2^ and ≥30% nucleated tumor cells required. DNA was extracted from macrodissected FFPE tissue. Molecular analysis used 20 ng of DNA with a commercially available NGS-targeted panel (TruSight Tumor 15, TST15, Illumina, San Diego, CA, USA) sequenced on the MiSeq platform (2 × 150 bp configuration, Illumina). The TST15 includes regions of 15 genes covering hotspot variants, including single-nucleotide variants and small insertions/deletions in the *AKT1*, *BRAF*, *EGFR*, *ERBB2*, *FOXL2*, *GNA11*, *GNAQ*, *KIT*, *KRAS*, *MET*, *NRAS*, *PDGFRA*, *PIK3CA*, *RET,* and *TP53* genes. Bioinformatic analysis used MiSeq Reporter with a manufacturer-supplied TST analysis module (Illumina). Variants were classified according to Sukhai et al. [11].

Baseline demographics, including age, sex, smoking status, stage and pathologic subtype, as well as treatment and clinical outcomes, were recorded prospectively. For each specimen tested, the type of sample and site of origin were identified.

Synchronous tumors were defined by repeat testing within 4 months from different tumors. Metachronous tumors were defined by repeat testing more than 4 months later on a different tumor. Unifocal (single) samples had multiple diagnostic samples taken from the same tumor site within 4 months.

## 3. Results

### 3.1. Patients and Samples

Between February 2017 and October 2020, 38 patients were identified to have multiple NGS results from a total of 82 lung carcinoma samples. The baseline and sample characteristics are listed in Table 1 and Table 2, respectively. The median age of patients was 66 years, 55% were women, 34% were lifetime never-smokers, and 97% had adenocarcinoma. Of the 82 samples analyzed, 66 samples (80.5%) were from patients with 2 samples tested, 12 (14.6%) from patients with 3 samples tested, and 4 (4.9%) from 1 patient with 4 samples tested A total of 11% had repeat testing on a single sample (unifocal), 51.2% on synchronous samples, and 37.8% on metachronous tumor samples. Eighty-three percent of the samples were obtained from the primary lung cancer site, and half of the samples tested were from core biopsies.

### 3.2. Synchronous vs. Metachronous Genotyping

**Figure 1:**
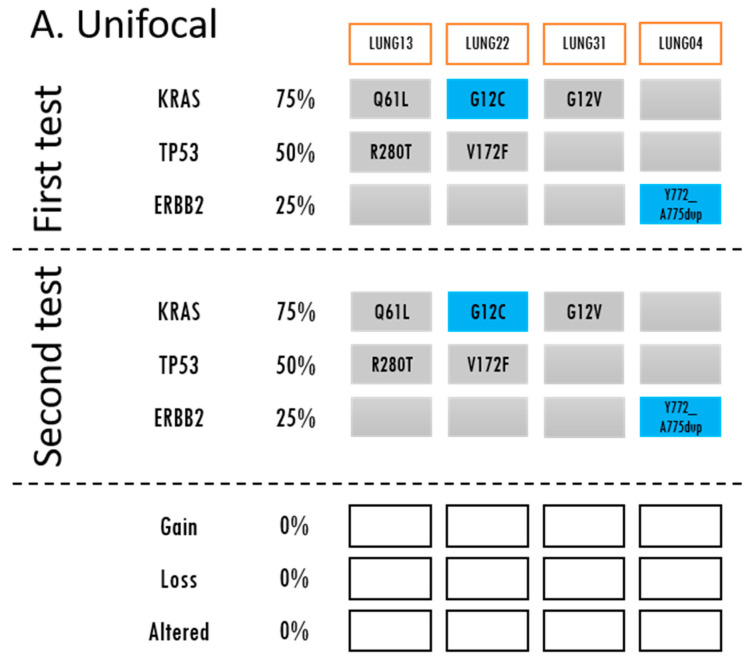

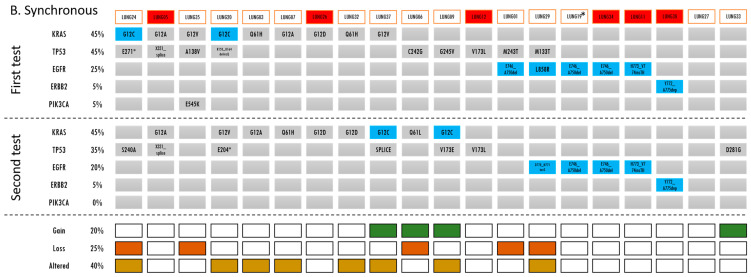

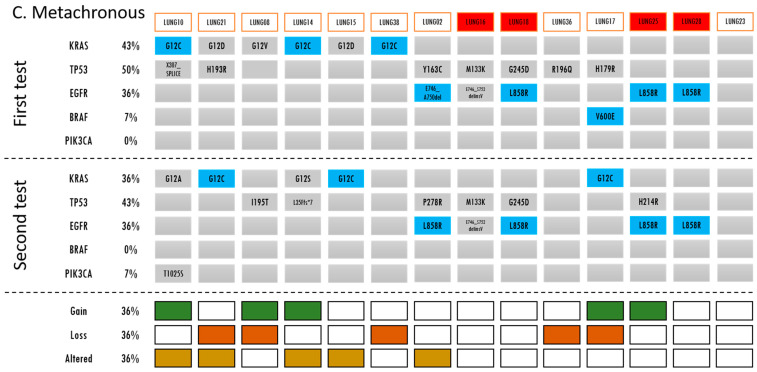
A blue box indicates that a driver mutation was detected in the gene of that sample. A grey box indicates that no currently actionable driver mutations were detected. The detected mutation is described in the box. If there were any gains, losses, or alterations in the mutations during the second molecular profiling, this is indicated by green, orange, and yellow, respectively. (**A**) Unifocal samples demonstrate no changes in gene variants. The first set samples are the biopsies and the second set samples are the matched resected tumors. (**B**) Synchronous samples had mutational alterations most commonly followed by losses, then gains. A red box around the subject ID indicates likely intrapulmonary metastasis. * For further information regarding LUNG19 (4 samples), please refer to Table S3. (**C**) For metachronous samples, the frequency of gains, losses and alterations were equal. A red box around the subject ID indicates likely metastasis.

Of 38 patients, 36 patients (94.7%) had at least one variant identified in their cancer sample using TST15. The most frequently identified variant was *TP53* (57.9%), followed by *KRAS* (55.3%) and *EGFR* (26.3%). Intra-patient changes in gene variants between samples were identified in 22 patients (58%) in *EGFR*, *KRAS*, *BRAF*, *PI3KCA*, and *TP53*. Among the single unifocal samples, there were no changes in variants identified between the NGS testing of the biopsy and NGS testing of the resected tumor from the same patient (Figure 1A).

However, differences in variants were reported in 60% of synchronous tumor samples and 71% of metachronous tumor samples, including gains, losses, or alterations in variants. Among synchronous samples (within 4 months of primary diagnosis), changes in single-nucleotide variants (SNVs) were most commonly seen (40%), followed by SNV losses (25%) and SNV gains (20%). Of 20 patients with tested synchronous tumor samples, clinically relevant alterations were present in one sample but not the other for six patients (LUNG24, 20, 37, 09, 01, 29). These included *KRAS* G12C (N = 4; LUNG24, 20, 37, 09), *EGFR* ex19del (N = 1; LUNG01), and *EGFR* L858R in one sample and an *EGFR* ex20ins mutation in another (N = 1; LUNG29) (Figure 1B). LUNG37 received immunotherapy based on PD-L1 tumor expression and a lack of access to KRAS targeted therapy at the time. Others had early-stage disease and were not candidates for targeted therapy during the study period.

For metachronous tumor samples, the frequencies of gains, losses, and alterations were similar at 36% each. (Figure 1C). Five of fourteen patients (LUNG10, 14, 38, 02, 17) had AGAs detected in their primary diagnostic sample but not in their metachronous sample (3 *KRAS* G12C, 1 *EGFR* ex19del, 1 *BRAF* V600E). LUNG10 received chemotherapy and immunotherapy (no AGA in most recent sample), while the other four patients received no systemic therapy (early-stage disease). Another patient (LUNG02) had different EGFR mutations detected in the primary (ex19del) and metachronous lesions (L858R). This patient had recently started osimertinib with response. Repeat sampling for cases LUNG10, 14, 38, 17, and 2 occurred at 441, 513, 800, 1212, and 3127 days after the first sample. Due to early-stage disease and a lack of available targeted therapy, we were unable to assess the impact of discovering these incremental AGAs on treatment outcomes.

### 3.3. Patient Outcomes

Additionally, we took a more in-depth look at the synchronous samples that were deemed separate primaries (n = 8) versus intrapulmonary metastasis (n = 6) based on the genomic results. The median follow-up for these patients was 46.1 months (range: 1.9–77.5). In Figure S1, there is a significant difference in OS between the two groups (log rank *p* = 0.02). The median OS was 30.0 months (95% CI 10.5-NR) in the metastatic group. No deaths were observed in the separate primary group. The 3-year survival rates were 44.4% in the metastatic group (95% CI 16.7–100) and 100% for the separate primary group. The changes among samples per patient can be viewed in the Supplementary Materials.

## 4. Discussion

Repeat NGS testing, even with a limited 15-gene panel, identified differences in gene variants in more than 50% of patients with NSCLC with synchronous and metachronous tumors. In the case of synchronous tumors, we identified differences in 60% of patient samples, suggesting that these represent multiple primary lung carcinomas, while the other 40% likely represent intrapulmonary metastasis. We also identified differences in 71% of the metachronous samples, including new primary tumors. Some of these harbored actionable alterations, suggesting that some patients may benefit from repeat NGS testing of not only synchronous pulmonary lesions but also metachronous lesions, including metastatic recurrence. Despite the small sample size, we were able to identify a significant survival difference between patients deemed to have metastatic tumors and patients deemed to have synchronous primary lung cancers based on our limited NGS classification integrated into pathology assessment.

In clinical practice, differentiating between multiple primary lung cancers and intrapulmonary metastasis is challenging using pathology or morphologic appearance alone, and NGS is now recommended to help definitively differentiate these entities [5,8,9,10,11,12,13,14,15]. When using genomic results to define multiple primaries versus intrapulmonary metastasis, we observed significantly improved survival in those with genome-defined multiple primaries versus intrapulmonary metastasis. When multiple pulmonary nodules are identified clinically, multi-gene-panel NGS profiling should be considered to distinguish separate primary lung cancer from intrapulmonary metastasis [5,15].

Our study is limited by several factors, including sample size, the use of a single institution, and the limited NGS panel. Our study cohort predated routine funding of larger panel comprehensive NGS. However, our findings are consistent with those of previous reports of distinct genomic features of synchronous primary tumors versus metastases with broader panels [5,15]. Chang et al. (2019) focused on synchronous resected tumor pairs, demonstrating the importance of large NGS panel testing in differentiating multiple primary tumors from intrapulmonary metastasis. Most of the actionable driver alteration AGAs captured with the larger NGS panel reported by Chang et al. were also tested with the TST15 panel in our study. *ALK* and *ROS1* fusions were not included in the scope of our study, as these were not included in the TST15 panel, are less frequent, and were routinely tested via IHC at our institution during the study period. Yang et al. (2023) recently published a molecular classification system using NGS to identify separate pulmonary carcinomas from intrapulmonary metastasis. The presence of shared driver alterations, ≥1 nondriver somatic alteration, and similar TP53 alterations confirm metastasis, while discordance suggests a distinct primary tumor. Our cohort had a greater proportion of former or current smokers (65%) compared to 31% in the study by Yang et al. As anticipated, a greater proportion of KRAS mutations (55% versus 6%) and fewer EGFR mutations (26% versus 74%) were identified in our cohort [6]. In an extensive review of the literature, Chang and Rekhtman (2024) determined that the NGS-directed identification of distinct AGAs may distinguish between separate primaries and intrapulmonary metastasis in up to 80% of cases [15]. Our findings are compatible with those of this review, despite using the limited DNA-based panel available during the study period. While large NGS panels are preferred, not all countries can afford this technology; thus, our findings may be relevant to settings with limited NGS access.

While Chang et al. 2019 [5] focused on synchronous resected tumor pairs, our study provides further insight into metachronous tumors and highlights the importance of repeat NGS testing at the time of metastatic recurrence given the therapeutic implications. The importance of repeat biopsy in relapsed NSCLC may be helpful in several clinical scenarios. In patients with *EGFR*-mutated lung cancer, T790M mutations, *MET* amplifications, and other AGAs are among the acquired mechanisms of resistance to prior EGFR therapies, allowing for therapeutic options beyond chemotherapy. Liquid biopsy has also provided an easier means of obtaining genomic information, especially upon resistance, although sensitive detection of copy number variants, including *MET*, fusions, and diagnosis of small-cell transformation, still require tumor tissue. Some have also suggested repeat molecular testing in cases where the recurrence is more than 6 months from the initial diagnosis, even in those without AGAs identified in the diagnostic sample [12]. Our patients with new clinically relevant alterations in repeat tumor samples experienced recurrent disease between 441 and 3127 days after initial diagnosis. Sampling heterogeneity and potentially increased diversity in later stages of tumor progression, related to the evolution of subclones, may also play a role [13].

In our study, we identified five patients with repeat biopsies and *KRAS* G12C mutations identified in a subsequent tumor sample (three metachronous, two synchronous). This could allow patients to receive targeted therapy for up to 21% of metachronous and 10% of synchronous lesions. Our study is limited by the lack of targeted therapy access for these described cases. Thus, the impact on patient treatment outcomes of testing multiple samples is unknown from our study, but we believe it deserves further exploration.

The finding of emergent *EGFR* and *ALK* mutations in patients who progress on EGFR or ALK inhibitors supports the claim that driver mutations may be subclonal. In our study, two patients had repeat tumor biopsies with discordant *KRAS* G12C results. This loss of *KRAS* G12C could be due to the sensitivity threshold of the NGS assay or, alternatively, a reversion to the wild type, which has also been described in colorectal cancer, whereby loss of this mutation is a mechanism the tumor uses to escape immune detection [16]. Thus, it appears that some patients may benefit from repeat biopsy and repeat genomic testing on metastatic recurrences, although further data on clinical outcomes with targeted therapy are required.

## 5. Conclusions

Although our study at a single institution is limited, our findings suggest that repeat NGS testing in patients with synchronous and metachronous NSCLC may yield important clinical information. Clearly, genomic testing is of growing importance in differentiating multiple primary lung cancers from intrapulmonary metastasis, although our understanding of potential confounding through genomic heterogeneity is limited. In addition, repeat biopsy and sampling of metastatic recurrence may identify clonal heterogeneity and evolution in metastasis, including the potential for identifying novel actionable genomic alterations. This may lead to differing therapeutic decisions by identifying changes in specific gene variants in recurrent versus primary tumor samples. As more targetable alterations are discovered and treatments are developed for early and late-stage NSCLC, this will become even more relevant for clinical practice. Larger studies will be required to generate definitive recommendations for clinical practice.

## Figures and Tables

**Table 1:** Patient and disease characteristics (N = 38).

	All Patients N = 38
Age at diagnosis, median (range)	66 years (47–86)
Sex	
Female	21 (55.3%)
Male	17 (44.7%)
Smoking status	
Never	13 (34.2%)
Former	19 (50.0%)
Current	6 (15.8%)
Stage at diagnosis	
I	16 (44.4%)
II	1 (2.8%)
III	2 (5.6%)
IV	6 (16.7%)
Multiple primaries	11 (30.6%)
Unknown	2
Histology	
Adenocarcinoma	37 (97.4%)
Squamous	1 (2.6%)

**Table 2:** Sample characteristics (N = 82 samples).

	Number (%) N = 82
Samples tested per patient *	
2	66 (80.5%)
3	12 (14.6%)
4	4 (4.9%)
Sample type	
Core biopsy	41 (50.0%)
Surgical specimen	39 (47.6%)
Exfoliative cytology	1 (1.2%)
FNA cytology	1 (1.2%)
Sample site	
Primary (lung)	68 (82.9%)
Non-bone visceral or soft tissue metastasis	11 (13.4%)
Bone metastasis	2 (2.4%)
Pleural fluid	1 (1.2%)
Sample timing	
Repeated **	9 (11.0%)
Synchronous (<4 months)	42 (51.2%)
Metachronous (>4 months)	31 (37.8%)

* Sample IDs for patients with 2, 3 and 4 samples are listed in Tables S1, S2 and S3 respectively. ** Samples taken from biopsy followed by resection of same tumor.

## Data Availability

The data presented in this study are not publicly available due to the privacy of individuals. The data presented in this study may be made available upon reasonable request from the senior author.

## Supplementary Materials

The following supporting information can be downloaded at: https://www.mdpi.com/article/10.3390/curroncol31080334/s1, Figure S1: Comparison of overall survival of synchronous separate primary lung cancer (n = 8) versus synchronous metastasis (n = 6); Table S1: List of patients with two samples tested (n = 32); Table S2: List of patients with three samples tested (n = 5); Table S3: List of patients with four samples tested (n = 1).
