# Supplementary material for: Repeat Next-Generation Sequencing (15-Gene Panel) in Unifocal, Synchronous, and Metachronous Non-Small-Cell Lung Cancer—A Single-Center Experience
Source: Curr Oncol. 2024 Aug 3;31(8):4476–85. doi: 10.3390/curroncol31080334 (PMC11352737; doi:10.3390/curroncol31080334)
Supplement: Supplementary file 1 [file curroncol-31-00334-s001.zip › curroncol-3067967-supplementary.pdf]

Supplementary Materials

Figure S1. Comparison of overall survival of synchronous separate primary lung cancer (n=8) versus synchronous metastasis (n=6).

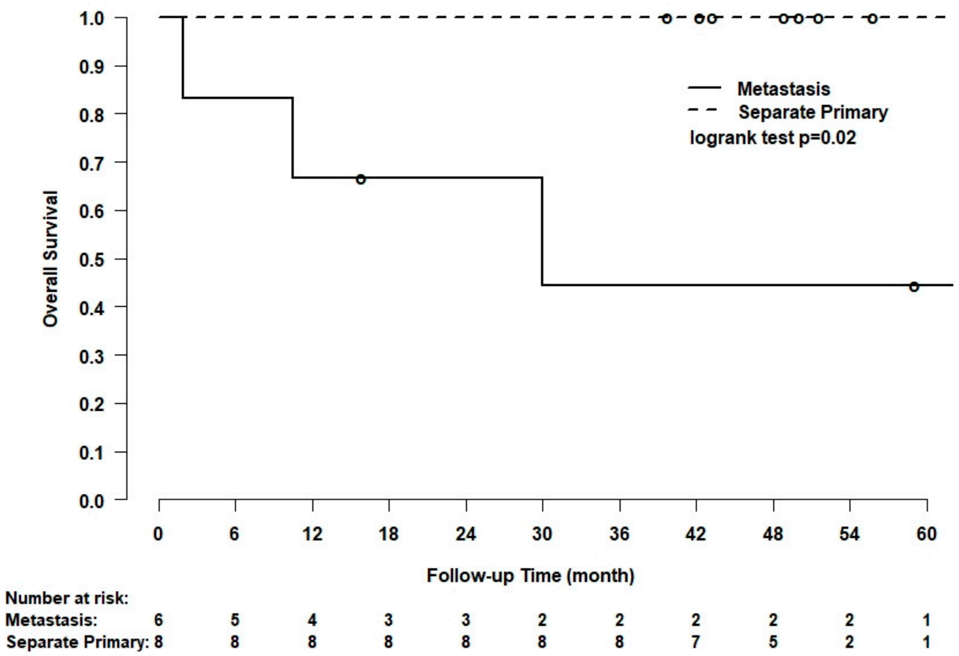

Table S1. List of patients with two samples tested (N=32).

Sample ID	Sample Site 1	Gene Variant 1	Sample Site 2	Gene Variant 2	Comparison	Timing	Days Between Sample Reports	Interpretation of NGS results	Treatment preceding second biopsy*
LUNG31	Lung Biopsy	*KRAS* c.35G>T (p. Gly12Val) 28%	Lung Resection	*KRAS* c.35G>T (p.Gly12Val) 7%	No change	Repeated	47	Same primary	None
LUNG04	Bone Cytology	*ERBB2* c.2313_2324dupATACGTGATGGC (p.Tyr772_Ala775dup) 45%	Bone Biopsy	*ERBB2* c.2313_2324dupATACGTGATGGC (p.Tyr772_Ala775dup) 12%	No change	Repeated	68	Same primary	None
LUNG22	Lung Biopsy	*KRAS* c.34G>T (p.Gly12Cys) 52%<br>*TP53* c.514G>T (p.Val172Phe) 77%	Lung Resection	*KRAS* c.34G>T (p.Gly12Cys) 25%<br>*TP53* c.514G>T (p.Val172Phe) 16%	No change	Repeated	20	Same primary	Immunotherapy
LUNG05	Lung Biopsy	*TP53* c.993+1G>T (p.?) 24%<br>*KRAS* c.35G>C (p.Gly12Ala) 24%	Lung Biopsy	*TP53* c.993+1G>T (p.?) 29%, c.844C>T (p.Arg282Trp)<br>*KRAS* c.35G>C (p.Gly12Ala) 22%	No change	Synchronous	0	Intrapulmonary metastasis	None
LUNG11	Lung Biopsy	*EGFR* c.2319_2320insACCCAC (p.His773_Val774insThrHis) 34%	Lymph Node Biopsy	*EGFR* c.2319_2320insACCCAC (p.His773_Val774insThrHis) 11%	No change	Synchronous	19	Metastasis (LN)	Chemotherapy/ Targeted
LUNG12	Lymph Node Biopsy	*TP53* c.517G>C (p.Val173Leu) 8%	Lung Biopsy	*TP53* c.517G>C (p.Val173Leu) 26%	No change	Synchronous	17	Metastasis (LN)	None
LUNG26	Lung Resection	*KRAS* c.35G>A (p.Gly12Asp) 18%	Lung Resection	*KRAS* c.35G>A (p.Gly12Asp) 3%	No change	Synchronous	63	Intrapulmonary metastasis	None
LUNG27	Lung Biopsy	None detected	Lung Biopsy	None detected	No change	Synchronous	0	Indeterminate	None
LUNG30	Lung Biopsy	*ERBB2* c.2313_2324dupATACGTGATGGC (p.Tyr772_Ala775dup) 55%	Lung Biopsy	*ERBB2* c.2313_2324dupATACGTGATGGC (p.Tyr772_Ala775dup) 28%	No change	Synchronous	104	Intrapulmonary metastasis	None
LUNG34	Lymph Node Biopsy	*EGFR* c.2235_2249delGGAATTAAGAGAAGC (p.Glu746_Ala750del) 56%	Lung Biopsy	*EGFR* c.2235_2249delGGAATTAAGAGAAGC (p.Glu746_Ala750del) 31%	No change	Synchronous	62	Metastasis (LN)	Targeted
LUNG01	Lung Biopsy	*EGFR* c.2235_2249delGGAATTAAGAGAAGC (p.Glu746_Ala750del) 42%<br>*TP53* c.728T>C (p.Met243Thr) 10%	Lung Resection	None detected	Loss	Synchronous	82	Separate primaries	None
LUNG29	Lung Biopsy	*EGFR* c.2573T>G (p.Leu858Arg) 83%<br>*TP53* c.398T>C (p.Met133Thr) 34%	Lung Resection	*EGFR* c.2310_2311insGGG (p.Asp770_Asn771insGly) 30%	Altered	Synchronous	56	Separate primaries	None
LUNG33	Lung Biopsy	None detected	Lung Resection	*TP53* c.842A>G (p.Asp281Gly) 14%	Gain	Synchronous	47	Separate primaries	None
LUNG32	Lung Biopsy	*KRAS* c.183A>T (p.Gln61His) 42%	Lung Biopsy	*KRAS* c.35G>A (p.Gly12Asp) 7%	Altered	Synchronous	16	Separate primaries	Chemotherapy
LUNG06	Lung Biopsy	*TP53* c.724T>G (p.Cys242Gly) 33%	Lymph Node Biopsy	*KRAS* c.182A>T (p.Gln61Leu) 6%	Loss/Gain	Synchronous	43	Indeterminate	None
LUNG07	Lung Biopsy	*KRAS* c.35G>C (p.Gly12Ala) 17%	Lung Resection	*KRAS* c.183A>C (p.Gln61His) 21%	Altered	Synchronous	58	Separate primaries	None
LUNG09	Lung Biopsy	*TP53* c.734G>T (p.Gly245Val) 34%	Lymph Node Biopsy	*TP53* c.518T>A (p.Val173Glu) 28%<br>*KRAS* c.34G>T (p.Gly12Cys) 25%	Gain	Synchronous	0	Indeterminate	Immunotherapy
LUNG24	Lung Biopsy	*TP53* c.811G>T (p.Glu271*) 11%<br>*KRAS* c.34G>T (p.Gly12Cys) 27%	Lung Resection	*TP53* c.720T>A (p.Ser240Arg) 30%	Loss	Synchronous	89	Indeterminate (favor separate primaries)	Chemotherapy
LUNG03	Lung Biopsy	*KRAS* c.183A>T (p.Gln61His) 32%	Lung Biopsy	*KRAS* c.35G>C (p.Gly12Ala) 12%	Altered	Synchronous	62	Separate primaries	None

LUNG35	Lung Resection	*PI3KCA* c.1633G>A (p.Glu545Lys) 9%<br>*KRAS* c.35G>T (p.Gly12Val) 16%<br>*TP53* c.413C>T (p.Ala138Val) 5%	Lung Resection	None detected	Loss	Synchronous	56	Separate primaries	None
LUNG37	Lung Resection	*KRAS* c.35G>T (p.Gly12Val) 5%	Lung Resection	*KRAS* c.34G>T (p.Gly12Cys) 13%<br>*TP53* c.559+1G>T (p.?) 9%	Gain	Synchronous	0	Separate primaries	None
LUNG16	Pleural Cytology	*TP53* c.398T>A (p.Met133Lys) 17%<br>*EGFR* c.2237_2255delAATTAAGAGAAG CAACATCinsT (p.Glu746_Ser752delinsVal) 48%	Lung Resection	*TP53* c.398T>A (p.Met133Lys) 37%<br>*EGFR* c.2237_2255delAATTAAGAGAAG CAACATCinsT (p.Glu746_Ser752delinsVal) 57%	No change	Metachronous	16	Clonal relation (same origin)	None
LUNG18	Lymph Node Biopsy	*TP53* c.734G>A (p.Gly245Asp) 28%<br>*EGFR* c.2573T>G (p.Leu858Arg) 73%	Liver Biopsy	*TP53* c.734G>A (p.Gly245Asp) 32%<br>*EGFR* c.2573T>G (p.Leu858Arg) 59%	No change	Metachronous	317	Clonal relation (same origin)	Chemotherapy
LUNG23	Lung Resection	None detected	Lung Resection	None detected	No change	Metachronous	777	Indeterminate, cannot rule out clonal relation (same origin)	None
LUNG28	Oral Cavity Biopsy	*EGFR* c.2573T>G (p.Leu858Arg) 91%	Lung Biopsy	*EGFR* c.2573T>G (p.Leu858Arg) 43%	No change	Metachronous	627	Clonal relation	None
LUNG25	Lung Biopsy	*EGFR* c.2573T>G (p.Leu858Arg) 14%	Liver Biopsy	*EGFR* c.2573T>G (p.Leu858Arg) 21%<br>*TP53* c.641A>G (p.His214Arg) 15%	Gain	Metachronous	624	Clonal relation[1] (heterogeneity)	None
LUNG02	Lung Biopsy	*EGFR* c.2236_2250delGAATTAAGAGAA GCA (p.Glu746_Ala750del) 8%<br>*TP53* c.488A>G (p.Tyr163Cys) 10%	Lung Resection	*EGFR* c.2573T>G (p.Leu858Arg) 43%<br>*TP53* c.833C>G (p.Pro278Arg) 55%	Altered	Metachronous	3127	Separate primaries	None
LUNG08	Lung Resection	*KRAS* c.35G>T (p.Gly12Val) 11%	Lung Resection	*TP53* c.584T>C (p.Ile195Thr) 19%	Loss/Gain	Metachronous	3119	Separate primaries	None
LUNG17	Lung Resection	*TP53* c.536A>G (p.His179Arg) 27%<br>*BRAF* c.1799T>A (p.Val600Glu) 22%	Lung Biopsy	*KRAS* c.34G>T (p.Gly12Cys) 14%	Loss/Gain	Metachronous	1212	Separate primaries	None
LUNG21	Lung Biopsy	*KRAS* c.35G>A (p.Gly12Asp) 20%<br>*TP53* c.578A>G (p.His193Arg) 20%	Lung Biopsy	*KRAS* c.34G>T (p.Gly12Cys) 19%	Loss	Metachronous	906	Separate primaries	None
LUNG36	Adrenal Resection	*TP53* c.587G>A (p.Arg196Gln) 37%	Lung Resection	None detected	Loss	Metachronous	941	Likely clonal heterogeneity	Chemotherapy
LUNG38	Lung Resection	*KRAS* c.34G>T (p.Gly12Cys) 11%	Lung Resection	None detected	Loss	Metachronous	800	Separate primaries	None

*Treatment preceding timing refers to treatment initiated within 30 days of the initial NGS report date.

[1]Clonal relation implies same origin and metastatic relationship.

Table S2. List of patients with three samples tested (N=5).

Sample ID	Sample Site 1	Gene Variant 1	Sample Site 2	Days Between Sample Reports	Gene Variant 2	Sample Site 3	Gene Variant 3	Comparison	Timing	Days Between Sample Reports	Treatment preceding 2nd+ biopsies
LUNG13	Lung Resection	*TP53* c.839G>C (p.Arg280Thr) 10% *KRAS* c.182A>T (p.Gln61Leu) 8%	Lung Resection	0	*TP53* c.839G>C (p.Arg280Thr) 55% *KRAS* c.182A>T (p.Gln61Leu) 53%	Lung Resection	*TP53* c.839G>C (p.Arg280Thr) 48% *KRAS* c.182A>T (p.Gln61Leu) 42%	No change	Repeated	13	None
LUNG15	Lung Biopsy	KRAS c.35G>A (p.Gly12Asp) 24%	Lung Resection	105	KRAS c.34G>T (p.Gly12Cys) 16%	Lung Resection	*KRAS* c.34G>T (p.Gly12Cys) 39%	Altered	Synchronous	574	None
LUNG20	Lung Biopsy	*TP53* c.473_490del GCGCCATG GCCATCTAC A (p.Arg158_Lys164delinsGln) 32% *KRAS* c.34G>T (p.Gly12Cys) 24%	Lung Resection	51	*TP53* c.610G>T (p.Glu204*) 16%	Lung Resection	*KRAS* c.35G>T (p.Gly12Val) 29%	Loss/Altered	Synchronous	63	None
LUNG10	Lung Biopsy	*KRAS* c.34G>T (p.Gly12Cys) 5%	Lung Resection	441	*TP53* c.919_919+1delinsAT (p.?) 16%	Lung Resection	*KRAS* c.35G>C (p.Gly12Ala) 29% *PI3KCA* c.3073A>T (p.Thr1025Ser) 33%	Gain/Altered	Metachronous	0	None
LUNG14	Lung Resection	*KRAS* c.34G>T (p. Gly12Cys) 18%	Lung Resection	513	*KRAS* c.35G>C (p.Gly12Ala) 33%	Lung Resection	*KRAS* c.34G>A (p.Gly12Ser) 37% *TP53* c.105_111del GCCGTCC (p.Leu35Phefs*7) 25%	Gain	Metachronous	536	None

Table S3. List of patients with four samples tested (N=1).

Sample ID	Sample Site 1	Gene Variant 1	Sample Site 2	Days Between Sample Reports	Gene Variant 2	Sample Site 3	Days Between Sample Reports	Gene Variant 3	Sample Site 4	Gene Variant 4	Comparison	Timing	Days Between Sample Reports	Treatment preceding 2nd+ biopsies
LUNG19	Lung Biopsy	*EGFR* c.2235_2249delGGAATTAAGAGAAGC (p.Glu746_Ala750del) 9%	Lung Resection	57	EGFR c.2235_2249delGGAATTAAGAGAAGC (p.Glu746_Ala750del) 15%	Lung Resection	0	EGFR c.2236_2250delGAATTAAGAGAAGCA (p.Glu746_Ala750del) 17%	Lung Resection	None detected	No change	Synchronous	115	None
